# Density-dependent recruitment but not survival drives cyclic dynamics in a field vole population

**DOI:** 10.1073/pnas.2509516122

**Published:** 2025-10-02

**Authors:** Xavier Lambin, Mike Begon, Sarah J. Burthe, Isla M. Graham, James L. MacKinnon, Sandra Telfer, Madan K. Oli

**Affiliations:** ^a^School of Biological Sciences, University of Aberdeen, Aberdeen AB24 2TZ, United Kingdom; ^b^Department of Evolution, Ecology and Behaviour, University of Liverpool, Liverpool L69 3BX, United Kingdom; ^c^Department of Wildlife Ecology and Conservation, University of Florida, Gainesville, FL 32611

**Keywords:** population cycles, demography, predation, voles, density dependence

## Abstract

Arguably, the most fundamental question in population ecology is what drives patterns in the abundance of populations? Small rodents exhibiting regular multiannual cycles in abundance have long been a test bed for addressing this question. The prevailing orthodoxy, the predation hypothesis, contends that nonmigratory, specialist predators are necessary, and specialist and generalist predators, combined, are both necessary and sufficient, for causing population cycles. Thus, variations in survival, from predation, are the key drivers of the cycles. However, this, and other competing theories, have hitherto lacked supportive demographic evidence and hence a solid evidential foundation. Here, we provide such evidence, analyzing 10 y of monthly data from a cyclic field vole population. We find, contrary to the prevailing orthodoxy, that recruitment, not survival, varied substantially from phase to phase in the cycles, made the major contribution to variations in population growth rate, and had cycle-phase-specific negative delayed density dependence. These results, their consistency with what is known from other systems, and the weak demographic foundations of the predation hypothesis, together suggest recruitment, specifically breeding-season length, not predation, as the cycles’ driving force. They therefore suggest that re-evaluation of the importance of the various determinants of population abundances, more generally, may be necessary.

## Significance

Ecologists seek to understand what drives patterns in the abundance of populations such as the multiannual cycles in abundance in rodents. The prevailing orthodoxy, the predation hypothesis, posits that changing patterns of predation-induced mortality cause population cycles. However, the prediction that variations in survival, from predation, should be the key demographic drivers of the cycles had hitherto not been tested because no suitable data existed. Here, we analyze 10 y of capture–recapture data from a cyclic field vole population. We find, contrary to the prevailing orthodoxy, that recruitment reflecting reproduction, not survival, varied substantially from phase to phase in the cycles, made the major contribution to variations in population growth rate, and had cycle-phase-specific negative delayed density dependence.

Small rodent populations experiencing multiannual cycles in abundance have long been a test bed for theories of how population abundance is determined generally (1, 2). Reaching consensus on underlying biotic mechanisms has, however, been difficult in the absence of the necessary demographic data (3, 4). Our limited demographic understanding has, paradoxically, been inferred from longitudinal studies of populations that experience irregular noncyclic outbreaks (5); field experiments have typically been conducted at spatial and temporal scales vastly smaller than those of the processes of interest (6–9), while experiments spanning the 3 to 4 y of a typical vole cycle have failed to substantially modify cyclic dynamics (7, 8). Accordingly, even the best supported hypotheses lack demographic foundations.

The prevailing orthodoxy is that population cycles arise because of variation in predation-induced mortality (10). This predation hypothesis postulates that nonmigratory, specialist predators (small mustelids, hereafter weasels) are necessary, and specialist and generalist predators, combined, are both necessary and sufficient, for causing population cycles (10–12). Supporting evidence has, however, mostly been indirect: either inference from statistical analyses of time series of abundance indices (13, 14) or circumstantial evidence from altered dynamics when weasel predation is more strongly linked to the voles (15) or altogether absent (16). Hence, while it is incontrovertible that predation is the proximate cause of death of most rodents (17), the contention that *variation* in predation-induced mortality is responsible for their population cycles remains weakly supported.

Beyond the prevailing orthodoxy, there are other potential drivers of small rodent cycles. First, it is unknown whether well-established cycle-phase-related changes in survival, litter size, age at maturation, and seasonal reproduction (e.g., refs. 1, 3, and 18), taken singly or together, are epiphenomena or are causally involved in generating cycles. Next, the voles’ strongly seasonal environment has been posited to interact with their variable, bimodal age at maturity (19, 20) in generating cycles (21), as has seasonally varying disease transmission (22–25). In particular, the timing of the onset of reproduction, at the end of plant winter quiescence, varies widely with cycle-phase, and hence with past population density (18, 26), leading to variation in the duration of exponential population growth during the breeding season^,^ and has a potentially large impact on population dynamics (3). However, a lack of appropriate data across all phases and seasons has precluded quantitative evaluation of its actual contribution (27) (but see ref. 28).

Thus, 101 y after Elton (29) described rodent population cycles, we still do not know the relative roles of survival, reproduction, and age of first reproduction in generating them. Different ecological processes are expected to leave distinct phase- and season-specific signatures on demographic rates: Variation in predation should be reflected in variation in prey (vole) survival, while changes in resource quality and availability should be reflected in variation in reproduction and recruitment.

Here, we analyze unprecedented data from a cyclic field vole (*Microtus agrestis*) population in Northern England, sampled monthly over three cycles. We use the temporal symmetry capture–mark–recapture model (30) to estimate and model apparent survival (*ϕ_t_*) and recruitment rates (*f_t_*) while accounting for imperfect detection. We characterize the syndrome of demographic change over cycles and test predictions from hypotheses invoking predation as a driver of population cycles.

## Results

### Cyclic Population Dynamics.

**Fig. 1.**
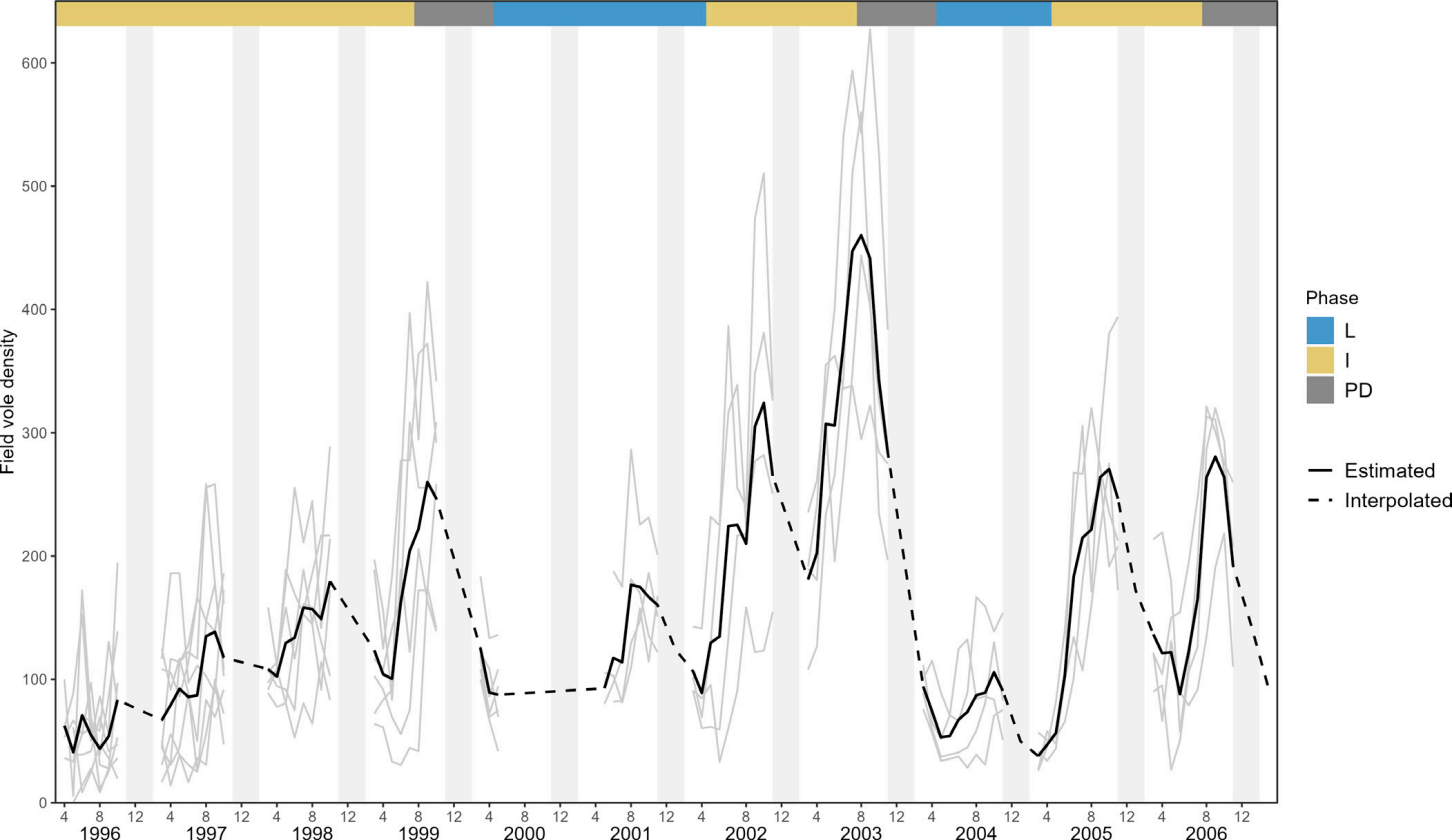
Field vole dynamics (voles/ha) in Kielder Forest, England (1996 to 2006) showing field vole densities (voles/ha) in individual trapping grids (thin gray lines), the average from 4 to 8 live-trapping grids contributing to the analyses (black line). Dotted lines show interpolated trajectories when the trapping intervals were more than 1 mo. Low (L) phase in blue, increase (I) in yellow, peak decline (PD) in dark gray. Light gray boxes show the 4 mo of winter, Nov–Feb.

Long-term monitoring revealed that the Kielder Forest field vole population exhibits cyclic dynamics with a 3 to 4-y periodicity and variable amplitude (Fig. 1). Our intensive study (March 1996 to January 2007) overlapped a period with dampened amplitude at the regional scale and increased spatial asynchrony between local populations (31, 32). Three multiannual cycles (with peak densities spanning 165 to 625 voles/ha) are recognizable, overlaying a clear seasonal component in the dynamics (Fig. 1). Based on the pattern of population fluctuation, we delineated a priori three cyclic phases: increase (I), peak-decline (PD), and low (L) to allow for the time dependency expected in cyclic populations (see Methods). Based on the biology of our study species, we divided a year into spring (SP; March–April), early summer (ES; May–July), late summer (LS; August–September), and fall-winter (WI; October–February). Our analyses consider 21,884 captures of 10,163 individual voles over 98 primary capture occasions.

**Fig. 2.**
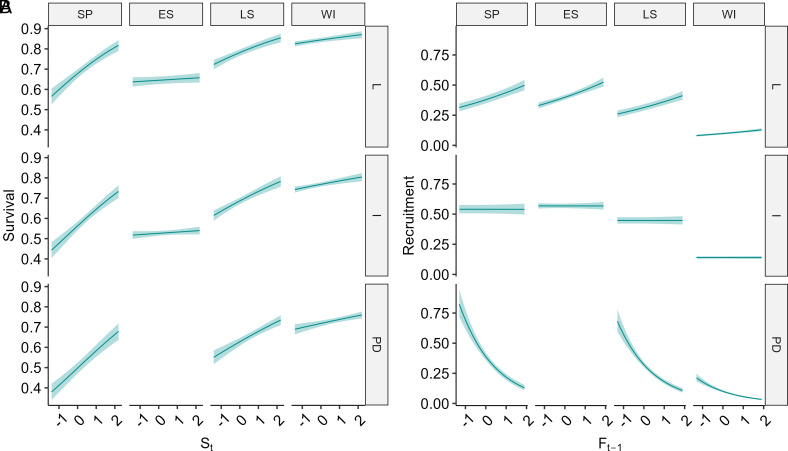
The influence of standardized current spring’s population density (S_t_) on survival (*A*) and of standardized previous autumn density (F_t−1_) on per capita recruitment rate (*B*) during each season and cyclic phase of field voles in Kielder Forest, England, 1996 to 2006, as predicted by model ϕ(season * S_t_ + phases)p(time)f(phases * F_t−1_ + season). Phases are I = increase; PD = peak-decline; and L = low. Seasons are SP = spring; ES = early summer; LS = late summer. Blue envelopes denote 95% confidence limits.

**Fig. 3.**
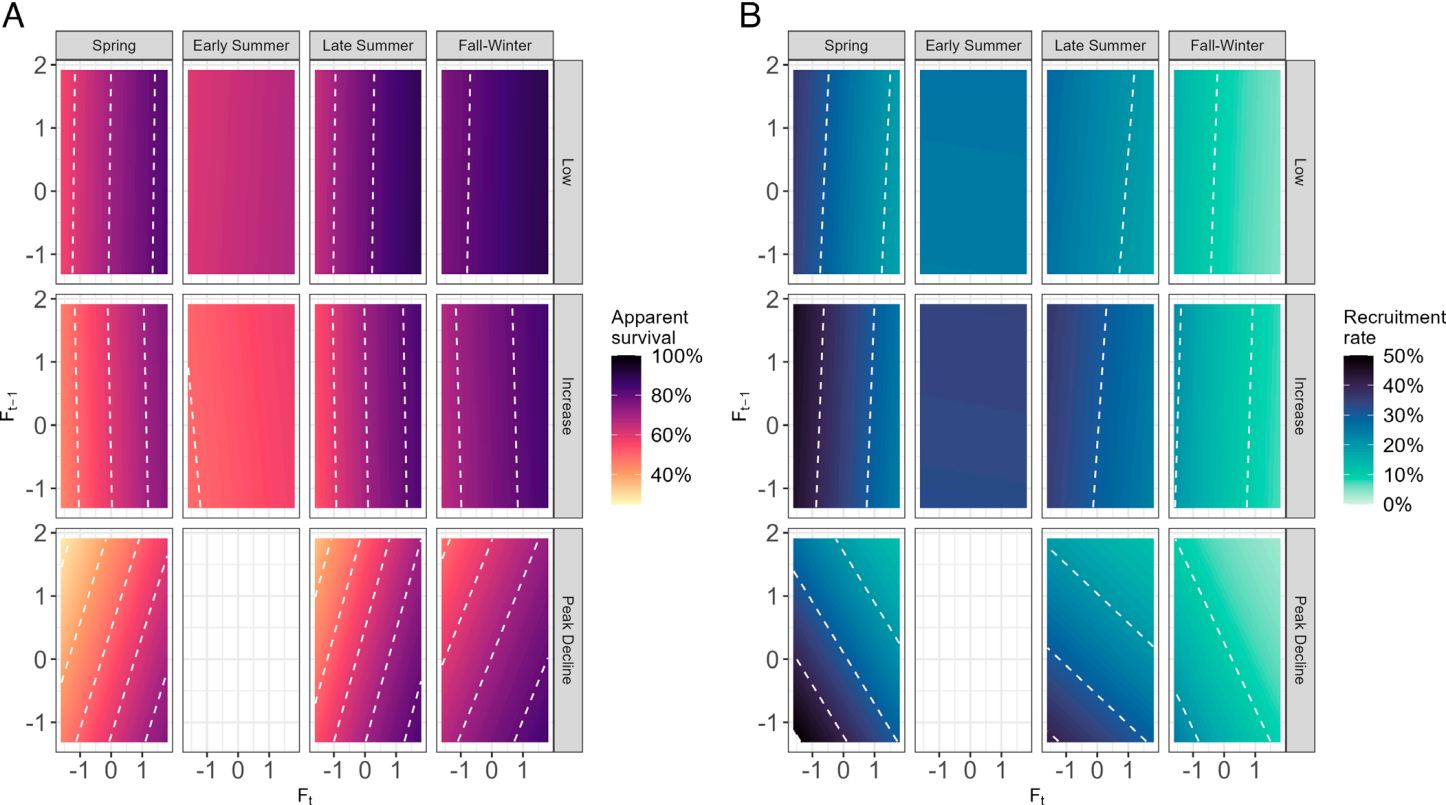
Contour plot showing predicted value of survival (*A*, *Left* 12 panels) and recruitment (*B*, *Right* 12 panels) from the best model ϕ(season * F_t_ + phases * F_t−1_)p(time)f(season * F_t_ + phases * F _t−1_). Each panel shows season-and cycle-phase-dependent survival and recruitment rates in relation to standardized recent and more distant past fall vole density. White dashed lines denote 10% increments in rates. Vertical color gradients and lines denote direct density dependence; horizontal color gradient and lines denote delayed density dependence. Tilted lines denote both direct and delayed density dependence.

Our stepwise analyses allowed increasing complexity in CMR models incorporating, in turn, only the effects of season and phase, then also a single current or lagged density covariate, and finally models with up to two density covariates and two-way interactions. The latter were substantially most parsimonious (Table 1), but we also illustrate predictions of models from the 2nd and 3rd steps here for clarity (Figs. 2 and 3) and provide all model coefficients in *SI Appendix*, Tables S1–S4.

**Table 1.** Models from three steps of analysis ranked by AICc showing: A. Models with seasons and phases only; B. Models with a single density covariate; C. Models with up to two density covariates

	Npar	AICc	∆AICc	Weight
A. Top four models with season plus phase or density				
ϕ(phases * season)p(time)f(phases * season)	120	127,454	0	1
ϕ(phases + season)p(time)f(phases * season)	115	127,489	35.32	0
ϕ(phases * season)p(time)f(phases + season)	115	127,507	53.62	0
ϕ(phases + season)p(time)f(phases + season)	110	127,555	101.57	0
B. Top four models with a single density covariate − ∆AICc from model set A: 210.9				
ϕ(season * S_t_ + phases)p(time)f(phases * F_t-1_ + season)	117	127,243	0	0.76
ϕ(phases * F_t_ + season)p(time)f(season * F_t_ + phases)	117	127,245	2.48	0.22
ϕ(season * phases + F_t_)p(time)f(phases * F_t_ + season)	119	127,250	7.11	0.02
ϕ(season * F_t_ + phases)p(time)f(phases * F_t_ + season)	117	127,261	18.55	0
C. Top four models with two density covariate models - ∆AICc from model set B: 64.3				
ϕ(season * F_t_ + phases * F_t-1_)p(time)f(season * F_t_ + phases * F_t-1_)	124	127,179	0	0.97
ϕ(season * S_t_ + phases * F_t-1_)p(time)f(phases * F_t_ + season * S_t-1_)	124	127,186	7.2	0.03
ϕ(season * S_t_ + phases + F_t-1_)p(time)f(phases * F_t_ + season * S_t-1_)	122	127,193	14.24	0
ϕ(phases * season + F_t_ + F_t-1_)p(time)f(phases * F_t-1_ + season * F_t_)	124	127,194	15.58	0

Npar denotes the number of estimated parameters, *ϕ* and *f*denote apparent monthly survival and recruitment rates respectively. p denotes trappability.

### Patterns of Variation in Survival.

CMR models show survival varied markedly among seasons, being highest in fall-winter followed by late summer, and also varied, though less markedly, with phase, being lowest in the peak-decline phase and highest in the low phase (Table 1*A* and *SI Appendix*, Fig. S1 and Tables S1–S3). In addition, models receiving strong support included positive direct density dependence on recent density (S_t_ or F_t_) interacting with season, being especially strong in spring and late summer (Fig. 2 and Table 1*B*). The most complex and best model also provided strong evidence of negative delayed density dependence (F_t−1_: lags 12 to 23 mo), though only during the peak-decline phase, starting in late summer and spanning the subsequent fall-winter and spring (Fig. 3 and Table 1*C*). Notably, however, the slopes of the positive direct density dependences were 1.5 times steeper than those of the negative delayed density dependences (*SI Appendix*, Table S3).

### Patterns of Variation in Recruitment.

As expected, given the known seasonality in vole reproduction, recruitment rate varied among seasons but, in contrast to survival, it also varied substantially among cycle phases (Figs. 2 and 3 and Table 1 B and C). Seasonally, recruitment was lowest in fall-winter when breeding is rare (range: 0.08 to 0.13 recruits per individual in the previous sampling occasion, per month), but then rose sharply in spring and early summer. Phase-specific variation in recruitment was itself strongest in spring, with the highest recruitment during the increase phase (0.59 recruits per individual per month; CI 0.57 to 0.61) and approximately halved recruitment during the peak-decline phase (0.26; CI 0.22 to 0.30; estimates from top model in Table 1*A* and *SI Appendix*, Fig. S1 and Table S1). These phase-specific differences in recruitment continued into the subsequent low phase, too, throughout the early summer, late summer, and fall-winter, albeit with decreasing magnitudes.

Models also provided strong evidence of negative density-dependent effects on recruitment, namely phase-specific delayed density-dependent effects (F_t−1_; Fig. 2, Table 1, and *SI Appendix*, Tables S2 and S3) as well as season-specific direct density-dependent effects (F_t,_; Fig. 3, Table 1, and *SI Appendix*, Table S3). These values of F_t,_ and F_t-1_ were estimated at fixed census dates such that the direct and delayed density dependent relationships with monthly recruitment estimates have variable time lag lengths. Recruitment declined with recent past fall density (F_t_) in the following winters (1 to 5 mo subsequently) and springs (5 to 6 mo subsequently). No direct density dependence was detectable by late summer (10 to 11 mo lag). The negative delayed density dependence in recruitment was only detectable during the peak-decline phase; there was no evidence in the low and increase phases.

### Population Growth Rate: Patterns and Components.

**Fig. 4.**
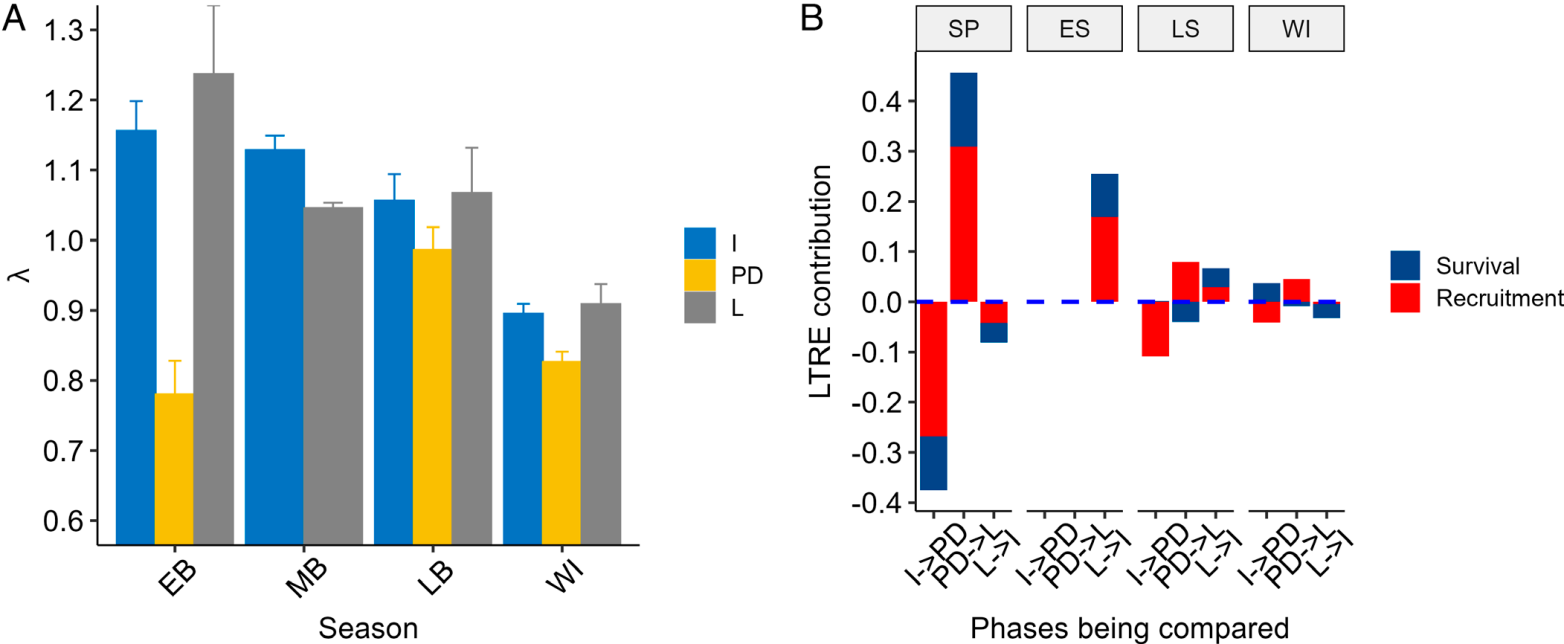
(*A*) Phase- and season-specific estimate of monthly realized population growth rate of field voles in Kielder Forest, England, 1996 to 2006, for each season and cyclic phase. (*B*) Lifetable response experiment contributions of recruitment (*f*) and survival (ϕ) to season and phase-specific differences in realized population growth rate. These analyses are based on the most parsimonious Pradel’s model without density covariates. The height of the bars is the sum of the *f* and ϕ contributions; the sum of these is the total observed change in realized population growth rate from one phase to the next. Phases are I = increase; PD = peak-decline; and L = low. Seasons are SP = spring; ES = early summer; LS = late summer, WI = fall-winter.

As expected from the seasonal- and phase-specific variation in survival and recruitment rates, the realized population growth rate, *λ*, also varied substantially seasonally, and, within each season, across cycle phases. The peak-decline phase was characterized by the lowest realized *λ* in all seasons, but this difference was particularly pronounced in spring (Fig. 4). Variation in *λ* was greatest in spring. Only in early summer was *λ* in the low phase lower than in the increase phase. There was limited phase variation in fall-winter *λ* (range *λ* = 0.834 − 0.901).

Changes in recruitment made substantially greater relative contributions (range 1.55 to 2.74-fold) to observed differences in *λ* across cyclic phases compared to changes in survival (Fig. 4). Similarly, the steep contrast in monthly *λ* in spring between the increase (*λ* = 1.15) and peak-decline phases (*λ* = 0.76) coincided with a 50% decline in recruitment but only an 18% reduction in survival. And the acceleration in spring growth from the peak-decline to the low phase, when populations grew at their fastest rate (*λ* = 1.24), reflected a 2.2-fold increase in recruitment but only a 1.3-fold improvement in survival. Growth differences in early summer between low (*λ* = 1.04) and increase (*λ* = 1.13) phases similarly reflected a 1.96-fold greater contribution from increased recruitment than improved survival.

## Discussion

Population ecologists have long been challenged with producing explanations for multiannual cycles in abundance that are supported by empirical evidence (1, 2, 33). The prevailing orthodoxy, grounded in theory, is that population cycles arise because of variation in predation-induced mortality rather than variation in reproduction (10), but it hitherto lacked demographic foundations. Our analyses of a uniquely large longitudinal data-set spanning all seasons and phases of three cycles, and at an appropriate sampling frequency, has enabled us to distinguish biologically distinct seasons from one another. Doing so has allowed us to provide estimates of the key demographic parameters and, of the importance of season, cycle phase and density in accounting for variation in those parameters.

Under the predation hypothesis, we expected i) a greater phase-specific variation in survival than in recruitment; ii) a greater contribution of survival than recruitment to phase-specific differences in realized population growth rate; and iii) a strongly negative effect of current and past population density on survival rates. Contrary to expectation i), we uncovered a consistent demographic syndrome dominated by substantial cycle-phase-related variation, around a common seasonal pattern, for recruitment but hardly so for survival. This consistency emerged despite substantial variation in the amplitude and in the detailed topology of the fluctuations and despite averaging density covariates between imperfectly synchronous pooled trapping sites. Further, contrary to expectation ii), the contributions to variation in population growth rate were more than twofold greater for changes in recruitment than they were for changes in survival. And contrary to the expectation that variation in predation rate should be reflected in variation in prey survival iii), it was recruitment that had phase-specific negative delayed density dependence and season-specific negative direct density dependence, whereas variation in survival was mostly seasonal, with a lesser influence of cycle phases.

The absence of any demographic signature of a predator numerical response is not paradoxical given field voles in Kielder Forest fluctuate cyclically with a 20-fold amplitude, with locally very high but rarely very low densities, inconsistent with deep depletion by predation. Similarly, there was some asynchrony between nearby sampling sites that should avert starvation by mobile predators such as weasels (thin gray lines in Fig. 1). It has long been recognized that asynchronous prey populations have a stabilizing influence on predator–prey interactions similar to that of alternative prey (34). Weasels only have a short numerical response to characteristic vole cycles in Kielder Forest, as they do in agro-steppes in Spain where asynchronously fluctuating conspecific or heterospecific rodents act as alternative prey (35–37). Predator satiation by a diverse resident vole–predator guild showing a functional but little numerical response to vole density (38) provides a plausible explanation for the unexpected result that survival was positively density dependent in all cycle phases and seasons except early summer.

Our observation that survival only marginally decreased during the winter of cyclic declines, whereas recruitment in spring varied substantially between phases, underpins our demographic interpretation of vole population cycles as driven by variation in the length of winters without reproduction rather than variation in predation-induced mortality. Over winter, populations always decline by around 10 to 17% monthly because animals are lost to death but not gained via reproduction. Estimates of survival probability of 0.75 to 0.78 per month in winter varied strikingly little between phases and likely reflected true rather than apparent survival since little movement occurs in that season (39, 40). Winters come to an end (early spring begins) with the inception of reproduction in 8 to 10-mo-old, overwintered females. Crucially, early spring recruitment rate varied widely in our data, explained, statistically, by the influence of cycle phase and of past density with a lag of approximately 18 mo. This is likely to reflect variation in the rate of resource acquisition. Indeed, the hypothesis that changes in the quality of grasses available in winter and spring that reflect past grazing and plant phenology has strong theoretical and some empirical support for our study area (24).

Recruitment, as a per capita rate, conflates the contributions of in situ reproduction and variation in early survival of juveniles up to the age when they become trappable (18 d old), but our previous work in the same system demonstrated strong delayed density dependence on the timing of the onset of reproduction in spring, with a delay in first reproduction longer than the length of gestation (24 d) for every additional 100 voles per hectare present in the previous spring (26). Thus, variation in recruitment in early spring unambiguously reflects variation in the onset of reproduction. Females born in spring reproduce either in their year of birth, as early as 1 mo old, or delay first reproduction until after their first winter, aged up to 9 mo old (20, 21). This wide variation around the second mode of the bimodal distribution is demographically potent, because it determines the length of the breeding season and the duration of the period of exponential growth. It is compounded by an early summer period optimal for reproduction, including reproduction by early-maturing spring-born females, with no evidence of any direct or delayed density dependence constraint on recruitment, and with fast-growing, diverse plant communities. In all cycle phases, populations grew at their fastest rates earlier in the breeding season (spring and early summer).

While snapshot census techniques, widely relied on for estimating abundance indices, ignore heterogeneity in capture probability (41) and lack precision for quantifying per capita population growth rate at low densities, we combined estimates of survival and recruitment to estimate *λ.* We found no detectable variation in per capita population growth rate between the low and increase phases, contradicting the views that high predator/prey ratios or predation-induced stress carrying over between generations delay population recovery following population declines (42). In the absence of demographic data spanning all phases of vole cycles, those beliefs have limited empirical grounding. Our study considered the direct influence of predation as envisaged by refs. 10–12, but not any nonlethal influence of predators on reproduction. However, evidence that voles modulate reproduction in response to predation risk is at best equivocal and evidence that it impacts dynamics is altogether lacking (43).

A crucial question, though, is whether variation in recruitment underpinned by variation in breeding season length as demographic driver of multiannual cycles applies generally, or at least more widely, to other small mammal systems, or whether, instead, our system is atypical, leaving the consensus around the specialist predation hypothesis essentially unchallenged. No definite answer can be given until similar work is carried out in other systems. But if the evidence that has been used to support the specialist predation hypothesis is neither definitive nor unequivocal, and if there is little or nothing we know about these other systems that is inconsistent with interpretations invoking variation in recruitment rather than survival, and especially if there are data from these systems that may be said to support such hypotheses, then, we contend, our characterization of the demographic basis for voles cycles in Kielder Forest may indeed be widely applicable to related cyclic systems. We argue below that nothing is known for other systems that is inconsistent with our hypothesis, and indeed there are data from other systems that support it.

A single study (44), based on lethal trapping rather than CMR, claimed that variation in survival rather than reproduction drove cyclic dynamics in a farmland vole population in Finland, but this study did not provide data-based estimates of season- and phase-specific demographic parameters. Wherever vole CMR demographic rate estimates exist, there are hints of a syndrome of demographic changes involving maturation and recruitment. Variation is most evident in late winter and early spring, hence affecting the duration of exponential growth during the breeding season (reviewed in refs. 1, 18, and 45. Also, using CMR-based analyses similar to those used here, of long-term field data spanning five snowshoe hare population cycles, Oli et al. (46) showed that precipitous declines in winter survival and reduced recruitment rates trigger the population crash; however, the transition from low to increase phase of the cycle was driven primarily by substantial increases in early summer recruitment. These results therefore further support the possibility that variation in recruitment is the primary demographic driver of small mammal population cycles. Further, our demographic analyses are easy to reconcile with the large body of time-series related studies using snapshot censuses of abundance, taken at fixed dates often constrained by spring snow melt (31). These stress season-specific sequential density dependence (15, 47, 48) and the importance of long winters (49), as we do. Further still, these census data imply occasional overwinter positive population growth, hence necessarily reproduction, and a strong signature of delayed density dependence between fall and spring surveys (15, 49). Our year-round data, unconstrained by snowmelt, indicate that this apparent variation in overwinter population growth between fall and spring censuses likely reflects variation in the prevalence of early spring reproduction by overwintered females before feasible census dates in northern latitudes, following snowmelt. Our analyses are, however, wholly inconsistent with interpretations of analyses of these time-series that invoke specialist mustelid predators as responsible for delayed density dependence, in the absence of data on such predators.

Ecological theory pertaining to population cycles has historically been dominated by mathematical models representing predator–prey interactions that abstract out seasonality (2, 11, 15). More recent theory starts with the alternation of seasons with high or low reproduction, a fact rather than an assumption for many organisms including small herbivores. It then considers, specifically, delayed-density-dependent reproduction season-length, as evidenced in this paper, either assumed or arising through grazing-induced changes in overwinter food quality or infection dynamics affecting age at maturity (23–26). Fundamentally, seasonality in reproduction and seemingly subtle variation in age at maturity are strongly destabilizing forces when population dynamics unfolds in a seasonal environment (22, 26).

Our study also highlights the important distinction between factors or processes that generate patterns in the dynamics of populations (cycles in our case) and those that may amplify the patterns (50). What are described as “cycles” are in fact perceptible cycles that can be observed against a background of demographic noise, and while a signal-generator may be both necessary and sufficient to create cycles, signal-amplifiers may also be necessary for those cycles to be discerned. Our detailed demographic approach has allowed us to identify delayed-density-dependent breeding-season length, categorically, as a signal-generator. By contrast, previous observational and even experimental approaches have frequently compared populations that have discernible cycles with those that do not, then identified processes that differentiate between the two, most often levels or types of predation, and then proposed those processes as being responsible for the cyclicity (16, 17). This implies that they are cycle-generators, and certainly fails to distinguish between whether they are generators or amplifiers. We would not argue, here or in population ecology generally, that signal-generators are more “important” than signal-amplifiers. But if population dynamics are to be understood, the distinction between the two must be recognized and processes correctly classified.

It is odd that it has taken 101 y since Elton (29) for a demographic characterization of vole population cycles to be provided. Hypotheses invoking predator-induced changes in survival have proliferated without data-based estimates of phase-specific demographic rates. They are now refuted. Instead, our inference of changes in reproduction, most evident during the transition from winter to spring, provides a unifying perspective consistent with all strands of evidence across locations with cyclically fluctuating populations. Those changes were long known to exist (18) but a key insight from our study has been to highlight their much larger contributions to population dynamics than changes in survival. The remaining challenge is to identify the ultimate process responsible for delayed density-dependent recruitment in spring (44). It remains possible that different specific processes cause a similar demographic syndrome in different ecosystems, but, we contend, with our new analyses, the search has been substantially narrowed.

## Supplementary Material

Appendix 01 (PDF)

## Data Availability

Data and code data have been deposited in Figshare (https://figshare.com/s/c115e0f08161e6e9bc30) (51).
