# Supplementary material for: Density-dependent recruitment but not survival drives cyclic dynamics in a field vole population
Source: Proc Natl Acad Sci U S A. 2025 Oct 2;122(40):e2509516122. doi: 10.1073/pnas.2509516122 (PMC12519211; doi:10.1073/pnas.2509516122)
Supplement: Supplementary file 1 — Appendix 01 (PDF) [file pnas.2509516122.sapp.pdf]

Supplementary Materials for

DENSITY-DEPENDENT RECRUITMENT BUT NOT SURVIVAL DRIVES CYCLIC DYNAMICS IN A FIELD VOLE POPULATION

Authors: Xavier Lambin, Mike Begon, Sarah J. Burthe, Isla M. Graham, James L. MacKinnon, Sandra Telfer, Madan K. Oli

Corresponding author: x.lambin@abdn.ac.uk

The PDF file includes:

Fig. S1
Tables S1 to S4

Supplementary Figure S1

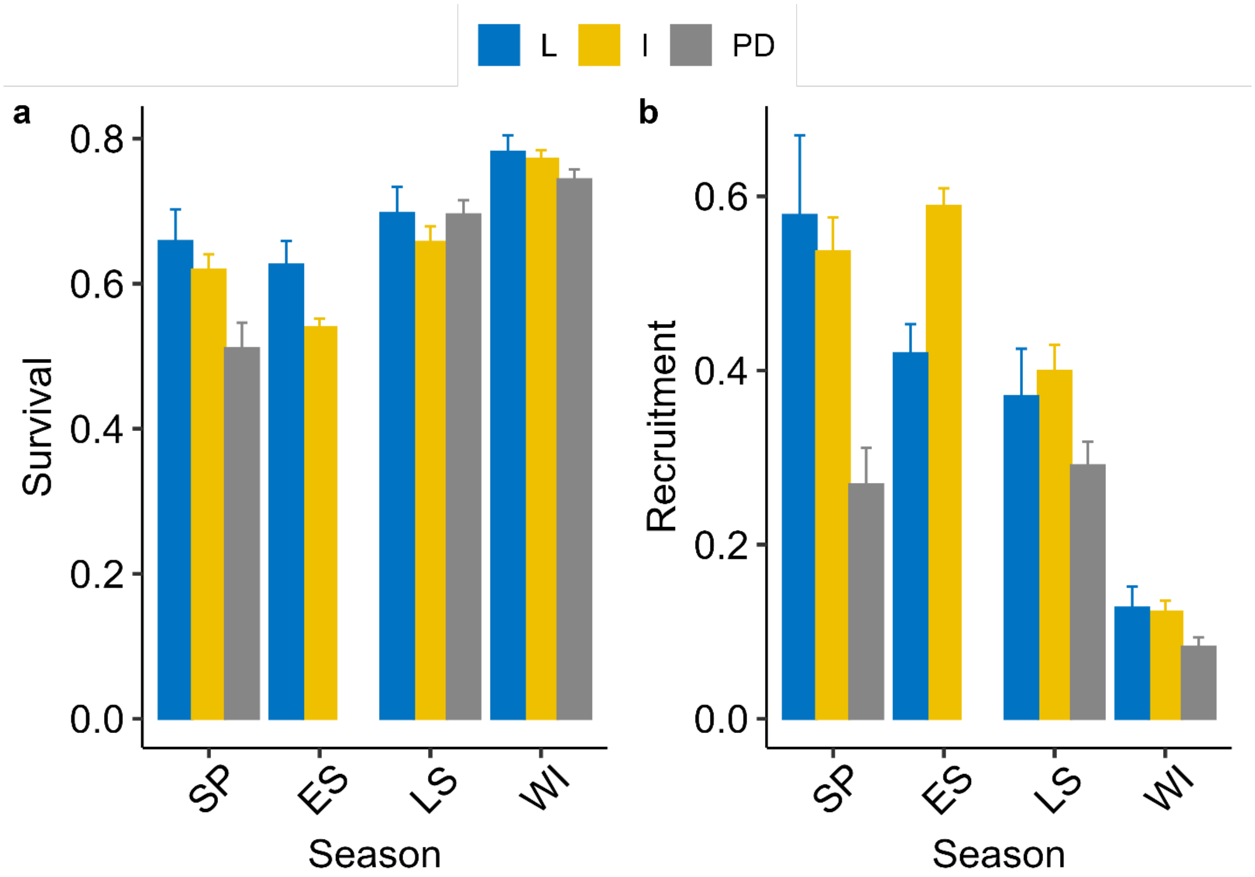

Figure S1. Phase- and season-specific estimates of monthly apparent survival and per capita recruitment rates of field voles in Kielder Forest, England, 1996-2006 predicted from model ϕ(phases * season)p(time)f(phases * season). Phases are: L = Low; I = Increase and PD = Peak-Decline. Seasons are: SP = Spring; ES = Early Summer; LS = Late Summer, WI =Fall-Winter.

Supplementary Tables S1 - S4

Table S1. Regression coefficients (β parameters) for the best model without density covariates (Table 1A), which included an interactive effect of phase and season on apparent survival (ϕ) and recruitment (f), and time effect on the capture probability (p). Phases are: L = Low; I = Increase and PD = Peak-Decline. Season s are: SP = Spring; ES = Early Summer; LS = Late Summer, WI =Fall-Winter. Spring is the reference Season and Increase the reference phase, which are included in the intercept term. A ":" symbol indicates interaction between the covariates involved. For example, (phase L:season ES) is the interaction between low phase and early spring season. Time indicates sampling occasions (1-137); 95% lcl and ucl refer to upper and lower confidence limits, respectively, and NA indicates inestimable parameter.

**Survival (ϕ)**	**estimate**	**se**	**95 % lcl**	**95 % ucl**
Intercept	0.485	0.047	0.393	0.577
phase PD	-0.441	0.085	-0.607	-0.274
phase L	0.172	0.109	-0.041	0.384
season MB	-0.326	0.053	-0.430	-0.221
season LB	0.166	0.069	0.032	0.301
season WI	0.736	0.060	0.618	0.854
phase L:season MB	0.185	0.132	-0.073	0.442
phase PD:season LB	0.615	0.110	0.399	0.831
phase L:season LB	0.011	0.151	-0.284	0.306
phase PD:season WI	0.286	0.104	0.082	0.490
phase L:season WI	-0.119	0.142	-0.397	0.159
**Recruitment (f)**	**estimate**	**se**	**95 % lcl**	**95 % ucl**
Intercept	-0.622	0.037	-0.694	-0.549
phase PD	-0.690	0.084	-0.855	-0.525
phase L	0.074	0.091	-0.104	0.252
season ES	0.092	0.045	0.005	0.180
season LS	-0.296	0.055	-0.403	-0.189
season WI	-1.473	0.065	-1.600	-1.346
phase L:season ES	-0.413	0.098	-0.606	-0.220
phase PD:season LS	0.373	0.104	0.170	0.576
phase L:season LS	-0.150	0.127	-0.400	0.100
phase PD:season WI	0.289	0.122	0.050	0.529

phase L:season WI	-0.039	0.149	-0.331	0.253
**Capture probability (p)**	**estimate**	**se**	**95 % lcl**	**95 % ucl**
(Intercept)	-0.232	0.223	-0.669	0.204
time 2	0.132	0.258	-0.374	0.638
time 3	0.820	0.316	0.199	1.440
time 4	0.915	0.334	0.261	1.570
time 5	-0.044	0.282	-0.598	0.509
time 6	-0.143	0.277	-0.685	0.400
time 7	0.665	0.296	0.085	1.244
time 12	2.799	0.801	1.228	4.370
time 13	2.413	0.481	1.470	3.356
time 14	1.556	0.357	0.857	2.255
time 15	1.498	0.367	0.778	2.218
time 16	1.461	0.332	0.810	2.112
time 17	2.096	0.370	1.371	2.820
time 18	2.194	0.361	1.487	2.901
time 19	1.499	0.348	0.817	2.180
time 24	15.422	NA	NA	NA
time 25	1.911	0.402	1.124	2.698
time 26	1.994	0.382	1.245	2.742
time 27	2.171	0.388	1.412	2.931
time 28	2.212	0.379	1.469	2.954
time 29	1.654	0.311	1.044	2.264
time 30	0.955	0.268	0.430	1.479
time 31	1.363	0.302	0.770	1.955
time 36	2.723	0.402	1.935	3.511
time 37	1.559	0.341	0.891	2.228
time 38	0.852	0.299	0.266	1.438
time 39	1.325	0.306	0.726	1.925
time 40	1.247	0.290	0.679	1.815

time 41	1.060	0.269	0.532	1.587
time 42	1.777	0.278	1.232	2.321
time 43	1.730	0.295	1.152	2.309
time 48	2.915	0.439	2.055	3.775
time 49	2.177	0.394	1.404	2.950
time 50	14.561	NA	NA	NA
time 62	-0.721	0.289	-1.287	-0.155
time 63	1.528	0.401	0.742	2.315
time 64	1.315	0.343	0.642	1.988
time 65	2.505	0.386	1.749	3.261
time 66	2.077	0.330	1.429	2.725
time 67	1.663	0.309	1.058	2.269
time 68	2.003	0.319	1.377	2.628
time 70	1.796	0.331	1.148	2.444
time 72	2.490	0.435	1.637	3.342
time 73	1.775	0.451	0.891	2.660
time 74	2.646	0.657	1.358	3.935
time 75	1.348	0.346	0.670	2.026
time 76	2.347	0.392	1.579	3.114
time 77	2.157	0.390	1.393	2.921
time 78	0.487	0.258	-0.018	0.991
time 79	1.537	0.277	0.995	2.079
time 80	1.112	0.256	0.611	1.614
time 81	1.128	0.257	0.624	1.632
time 83	0.921	0.258	0.416	1.427
time 85	1.697	0.289	1.131	2.262
time 86	1.720	0.298	1.136	2.305
time 87	2.874	0.379	2.132	3.616
time 88	2.054	0.315	1.437	2.671
time 89	2.935	0.398	2.155	3.715
time 90	1.858	0.271	1.327	2.390

time 91	1.491	0.255	0.990	1.992
time 92	1.578	0.253	1.083	2.073
time 93	1.595	0.261	1.083	2.106
time 94	1.545	0.266	1.023	2.066
time 96	2.225	0.342	1.555	2.895
time 98	1.677	0.432	0.830	2.523
time 99	2.650	0.835	1.014	4.287
time 100	1.401	0.426	0.567	2.236
time 101	2.018	0.615	0.813	3.223
time 102	1.904	0.532	0.862	2.946
time 103	1.947	0.486	0.995	2.900
time 104	1.861	0.434	1.010	2.712
time 105	0.679	0.316	0.060	1.299
time 106	0.609	0.310	0.002	1.217
time 107	-0.044	0.294	-0.621	0.533
time 109	-0.609	0.313	-1.223	0.004
time 111	0.228	0.348	-0.454	0.910
time 112	0.151	0.337	-0.510	0.812
time 113	0.820	0.363	0.108	1.532
time 114	2.331	0.478	1.395	3.267
time 115	2.856	0.428	2.017	3.695
time 116	2.449	0.364	1.736	3.163
time 117	2.234	0.364	1.521	2.947
time 118	3.005	0.426	2.170	3.840
time 119	1.933	0.296	1.352	2.515
time 120	1.734	0.286	1.173	2.295
time 122	1.710	0.299	1.125	2.295
time 124	1.928	0.343	1.255	2.601
time 125	2.477	0.451	1.593	3.361
time 126	3.230	0.902	1.462	4.999
time 127	1.032	0.354	0.339	1.725

time 128	1.746	0.434	0.895	2.597
time 129	1.288	0.324	0.652	1.924
time 130	1.880	0.331	1.230	2.529
time 131	2.264	0.307	1.662	2.867
time 132	2.397	0.317	1.776	3.017
time 133	1.483	0.283	0.929	2.037
time 135	2.297	0.380	1.553	3.042
time 137	49.631	NA	NA	NA

Table S2. Regression coefficients (β parameters) for the best model with a single density covariate (Table 1B), which included an interactive effect of season and spring density, and an additive effect of phase on apparent survival (ϕ); an interactive effect of phase and autumn density, and an additive effect of season; and time effect on the capture probability (p). Phases are: L = Low; I = Increase and PD = Peak-Decline. Seasons are: SP = Spring; ES = Early Summer; LS = Late Summer, WI =Fall-Winter. Spring is the reference Season and Increase the reference phase, which are included in the intercept term. A ":" symbol indicates interaction between the covariates involved. For example, (season LS:S_t) is the interaction between late summer season and spring density. Density covariates are: S_t = spring density, and F_t = autumn density. Time indicates sampling occasions (1-137); 95% lcl and ucl refer to upper and lower confidence limits, respectively, and NA indicates inestimable parameter.

**Survival (ϕ)**	**estimate**	**se**	**95 % lcl**	**95 % ucl**
Intercept)	0.253	0.044	0.165	0.340
season MB	-0.148	0.050	-0.246	-0.050
season LB	0.531	0.055	0.423	0.638
season WI	0.944	0.051	0.844	1.045
S_t	0.344	0.040	0.267	0.422
phase PD	-0.262	0.044	-0.348	-0.175
phase L	0.493	0.037	0.420	0.565
season ES:S_t	-0.320	0.046	-0.411	-0.228
season LS:S_t	-0.118	0.051	-0.218	-0.018
season WI:S_t	-0.246	0.047	-0.338	-0.153
**Recruitment (f)**	**estimate**	**se**	**95 % lcl**	**95 % ucl**
Intercept	-0.616	0.033	-0.681	-0.550
phase PD	-0.329	0.032	-0.393	-0.266
phase L	-0.355	0.026	-0.407	-0.304
F_{t-1}	0.000	0.010	-0.021	0.020
season ES	0.051	0.040	-0.028	0.129
season LS	-0.188	0.043	-0.274	-0.103
season WI	-1.353	0.051	-1.453	-1.253
phase PD:F_{t-1}	-0.577	0.045	-0.665	-0.490
phase L:F_{t-1}	0.145	0.026	0.094	0.196

Capture probability (p)	estimate	se	95 % lcl	95 % ucl
(Intercept)	-0.790	0.181	-1.145	-0.436
time 2	0.492	0.223	0.055	0.930
time 3	1.152	0.269	0.623	1.680
time 4	1.312	0.291	0.741	1.882
time 5	0.473	0.248	-0.014	0.960
time 6	0.323	0.240	-0.147	0.793
time 7	1.027	0.249	0.540	1.515
time 12	2.823	0.612	1.623	4.023
time 13	3.001	0.470	2.080	3.923
time 14	2.477	0.385	1.722	3.232
time 15	2.367	0.393	1.597	3.137
time 16	2.258	0.327	1.618	2.898
time 17	3.025	0.380	2.281	3.769
time 18	2.874	0.341	2.206	3.542
time 19	2.093	0.313	1.479	2.707
time 24	NA	NA	NA	NA
time 25	2.575	0.394	1.803	3.347
time 26	2.719	0.374	1.985	3.452
time 27	2.931	0.382	2.183	3.679
time 28	3.034	0.373	2.302	3.766
time 29	2.541	0.300	1.952	3.129
time 30	1.593	0.236	1.131	2.055
time 31	1.917	0.265	1.397	2.436
time 36	3.181	0.377	2.442	3.919
time 37	2.108	0.316	1.489	2.727
time 38	1.427	0.271	0.896	1.958
time 39	1.971	0.283	1.416	2.525
time 40	1.980	0.268	1.454	2.506
time 41	1.929	0.250	1.439	2.420
time 42	2.401	0.246	1.918	2.884

time 43	2.459	0.275	1.919	2.998
time 48	3.573	0.419	2.753	4.394
time 49	2.650	0.381	1.903	3.397
time 50	NA	NA	NA	NA
time 62	-0.359	0.252	-0.852	0.134
time 63	1.627	0.307	1.025	2.229
time 64	1.549	0.288	0.985	2.114
time 65	2.771	0.349	2.087	3.455
time 66	2.404	0.295	1.826	2.982
time 67	2.121	0.265	1.601	2.641
time 68	2.499	0.290	1.931	3.067
time 70	2.277	0.303	1.683	2.871
time 72	2.825	0.406	2.030	3.621
time 73	2.274	0.414	1.463	3.086
time 74	3.845	0.702	2.469	5.220
time 75	2.070	0.334	1.416	2.724
time 76	3.084	0.379	2.341	3.827
time 77	3.049	0.402	2.262	3.837
time 78	1.231	0.228	0.784	1.678
time 79	2.220	0.249	1.732	2.709
time 80	1.691	0.220	1.259	2.123
time 81	1.692	0.222	1.257	2.127
time 83	1.487	0.223	1.050	1.923
time 85	2.328	0.259	1.821	2.834
time 86	2.251	0.266	1.730	2.773
time 87	3.040	0.338	2.377	3.703
time 88	2.453	0.279	1.907	2.999
time 89	3.322	0.366	2.604	4.040
time 90	2.357	0.236	1.894	2.820
time 91	2.061	0.219	1.631	2.490
time 92	1.976	0.214	1.558	2.395

time 93	2.106	0.226	1.663	2.548
time 94	2.090	0.232	1.636	2.544
time 96	2.787	0.315	2.170	3.404
time 98	2.158	0.388	1.398	2.919
time 99	3.733	0.942	1.887	5.578
time 100	2.334	0.426	1.499	3.168
time 101	2.975	0.664	1.674	4.276
time 102	2.699	0.544	1.632	3.765
time 103	2.644	0.482	1.699	3.589
time 104	2.491	0.415	1.677	3.305
time 105	1.215	0.285	0.656	1.774
time 106	1.013	0.269	0.486	1.539
time 107	0.320	0.261	-0.191	0.831
time 109	-0.371	0.283	-0.925	0.183
time 111	0.133	0.292	-0.440	0.705
time 112	0.251	0.295	-0.326	0.829
time 113	1.113	0.324	0.478	1.748
time 114	2.644	0.441	1.779	3.508
time 115	3.354	0.408	2.554	4.154
time 116	3.006	0.341	2.337	3.674
time 117	2.923	0.352	2.233	3.613
time 118	3.601	0.407	2.802	4.400
time 119	2.501	0.266	1.980	3.021
time 120	2.305	0.255	1.805	2.806
time 122	2.279	0.269	1.752	2.805
time 124	2.536	0.319	1.912	3.161
time 125	3.059	0.432	2.211	3.907
time 126	3.776	0.889	2.034	5.518
time 127	1.676	0.340	1.010	2.342
time 128	2.520	0.451	1.637	3.404
time 129	2.040	0.309	1.435	2.645

time 130	2.934	0.340	2.268	3.601
time 131	2.559	0.269	2.032	3.086
time 132	2.988	0.288	2.423	3.553
time 133	2.081	0.252	1.587	2.575
time 135	2.929	0.359	2.225	3.633
time 137	NA	NA	NA	NA

Table S3. Regression coefficients (β parameters) for the best overall model with phase-specific and season-specific direct and delayed density dependence (Table 1C), which included an interactive effect of season and current autumn density, and an interactive effect of phase and previous autumn density on apparent survival (ϕ); an interactive effect of phase and current autumn density, and an interactive effect of season and previous autumn density on recruitment; and time effect on the capture probability (p). Phases are: L = Low; I = Increase and PD = Peak-Decline. Seasons are: SP = Spring; ES = Early Summer; LS = Late Summer, WI =Fall-Winter. Spring is the reference Season and Increase the reference phase, which are included in the intercept term. A ":" symbol indicates interaction between the covariates involved. For example, (season LS:F_t) is the interaction between late summer season and current autumn density. Density covariates are: F_t = current autumn density, and F_{t-1} = previous autumn density. Time indicates sampling occasions (1-137); 95% lcl and ucl refer to upper and lower confidence limits, respectively, and NA indicates inestimable parameter.

**Survival (ϕ)**	**estimate**	**se**	**95 % lcl**	**95 % ucl**
Intercept	0.417	0.043	0.333	0.501
season ES	-0.283	0.047	-0.376	-0.19
season LS	0.408	0.055	0.301	0.515
season WI	0.743	0.051	0.644	0.843
F_t	0.381	0.047	0.288	0.474
phase PD	-0.302	0.044	-0.388	-0.217
phase L	0.45	0.04	0.373	0.528
F_{t-1}	0.016	0.021	-0.025	0.058
season ES:F_t	-0.288	0.054	-0.394	-0.181
season LS:F_t	0.052	0.058	-0.061	0.165
season WI:F_t	-0.084	0.056	-0.193	0.026
phase PD:F_{t-1}	-0.257	0.047	-0.349	-0.166
phase L:F_{t-1}	-0.026	0.04	-0.104	0.053
**Recruitment (f)**	**estimate**	**se**	**95 % lcl**	**95 % ucl**
Intercept	-0.617	0.034	-0.683	-0.551
season ES	0.074	0.04	-0.005	0.153
season LS	-0.224	0.044	-0.309	-0.138
season WI	-1.297	0.05	-1.395	-1.199
F_t	-0.272	0.04	-0.35	-0.193

phase PD	-0.197	0.038	-0.272	-0.123
phase L	-0.404	0.029	-0.461	-0.347
F_{t-1}	0.021	0.014	-0.007	0.049
season ES:F_t	0.274	0.049	0.178	0.369
season LS:F_t	0.115	0.053	0.012	0.219
season WI:F_t	-0.08	0.067	-0.211	0.051
phase PD:F_{t-1}	-0.357	0.052	-0.459	-0.255
phase L:F_{t-1}	0.002	0.034	-0.065	0.07
**Capture probability (p)**	**estimate**	**se**	**95 % lcl**	**95 % ucl**
Intercept	-0.255	0.225	-0.696	0.186
time 2	0.35	0.274	-0.188	0.888
time 3	1.276	0.389	0.513	2.039
time 4	1.562	0.444	0.691	2.433
time 5	0.39	0.308	-0.214	0.995
time 6	0.217	0.294	-0.359	0.793
time 7	1.129	0.347	0.449	1.808
time 12	3.361	0.949	1.501	5.22
time 13	2.691	0.506	1.699	3.683
time 14	1.737	0.392	0.969	2.506
time 15	1.791	0.413	0.982	2.6
time 16	1.749	0.355	1.053	2.445
time 17	2.643	0.419	1.822	3.464
time 18	2.568	0.379	1.825	3.31
time 19	1.858	0.403	1.068	2.647
time 24	NA	NA	NA	NA
time 25	2.037	0.418	1.218	2.856
time 26	2.072	0.392	1.304	2.839
time 27	2.354	0.402	1.566	3.142
time 28	2.473	0.396	1.697	3.249
time 29	1.971	0.329	1.325	2.616
time 30	1.099	0.272	0.565	1.633

time 31	1.493	0.305	0.895	2.091
time 36	2.699	0.40	1.915	3.484
time 37	1.643	0.348	0.96	2.326
time 38	0.935	0.306	0.335	1.534
time 39	1.504	0.318	0.882	2.127
time 40	1.508	0.304	0.913	2.104
time 41	1.445	0.288	0.881	2.01
time 42	1.917	0.282	1.365	2.469
time 43	1.869	0.301	1.28	2.458
time 48	3.028	0.44	2.166	3.89
time 49	1.897	0.389	1.134	2.66
time 50	2.157	0.616	0.949	3.365
time 62	-1.084	0.282	-1.637	-0.531
time 63	0.828	0.312	0.216	1.44
time 64	0.901	0.309	0.296	1.506
time 65	2.286	0.378	1.546	3.027
time 66	2.043	0.331	1.395	2.692
time 67	1.644	0.302	1.053	2.235
time 68	1.964	0.32	1.338	2.591
time 70	1.722	0.331	1.072	2.372
time 72	2.226	0.425	1.393	3.06
time 73	1.619	0.424	0.788	2.451
time 74	2.873	0.68	1.541	4.205
time 75	1.35	0.347	0.67	2.03
time 76	2.371	0.396	1.594	3.148
time 77	2.288	0.408	1.488	3.088
time 78	0.603	0.265	0.083	1.123
time 79	1.591	0.283	1.037	2.145
time 80	1.061	0.256	0.559	1.564
time 81	1.095	0.259	0.588	1.602
time 83	0.91	0.26	0.401	1.419

time 85	1.657	0.291	1.087	2.227
time 86	1.665	0.297	1.082	2.247
time 87	2.885	0.38	2.139	3.63
time 88	2.013	0.314	1.398	2.627
time 89	2.855	0.394	2.083	3.626
time 90	1.832	0.271	1.30	2.364
time 91	1.488	0.256	0.985	1.991
time 92	1.466	0.252	0.973	1.959
time 93	1.526	0.262	1.013	2.038
time 94	1.558	0.269	1.031	2.085
time 96	2.364	0.346	1.687	3.042
time 98	2.37	0.518	1.355	3.385
time 99	3.192	0.938	1.354	5.03
time 100	1.592	0.439	0.732	2.452
time 101	2.216	0.646	0.951	3.482
time 102	2.002	0.542	0.939	3.064
time 103	1.942	0.484	0.993	2.89
time 104	1.694	0.41	0.891	2.498
time 105	0.488	0.299	-0.098	1.073
time 106	0.53	0.302	-0.063	1.122
time 107	-0.124	0.295	-0.702	0.454
time 109	-0.728	0.316	-1.347	-0.109
time 111	-0.031	0.339	-0.696	0.634
time 112	0.056	0.34	-0.61	0.722
time 113	0.975	0.397	0.197	1.752
time 114	2.479	0.5	1.499	3.459
time 115	2.929	0.431	2.084	3.775
time 116	2.536	0.369	1.813	3.258
time 117	2.511	0.387	1.752	3.269
time 118	3.076	0.434	2.226	3.926
time 119	1.885	0.295	1.306	2.464

time 120	1.714	0.287	1.151	2.276
time 122	1.7	0.3	1.112	2.287
time 124	1.906	0.343	1.233	2.578
time 125	2.464	0.449	1.583	3.345
time 126	3.214	0.891	1.468	4.961
time 127	1.022	0.351	0.334	1.711
time 128	1.751	0.431	0.906	2.597
time 129	1.36	0.327	0.718	2.002
time 130	2.218	0.358	1.517	2.919
time 131	2.031	0.3	1.443	2.619
time 132	2.402	0.318	1.779	3.024
time 133	1.546	0.286	0.985	2.106
time 135	2.408	0.384	1.654	3.161
time 137	NA	NA	NA	NA

Table S4. Model comparison statistics for models testing for appropriate model structure for capture probability (p). Parameters are: ϕ = apparent survival probability; f = recruitment rate; and p = capture probability. Phases are: L = Low; I = Increase and PD = Peak-Decline. Seasons are: SP = Spring; ES = Early Summer; LS = Late Summer, WI =Fall-Winter. A "+" indicates additive effect, "*" indicates additive and interactive effects. The number of parameters (K), Akaike information corrected for small sample size (AICc), difference in AICc (ΔAICc), model weight and deviance are also given.

**model**	**K**	**AICc**	**ΔAICc**	**weight**	**Deviance**
ϕ(phase*season)p(time)f(phase*season)	120	127453.695	0.000	1.000	7948.815
ϕ(phase+season)p(time)f(phase*season)	115	127489.016	35.320	0.000	7994.238
ϕ(phase*season)p(time)f(phase+season)	115	127507.316	53.620	0.000	8012.544
ϕ(phase+season)p(time)f(phase+season)	110	127555.271	101.575	0.000	8070.605
ϕ(phase*season)p(season*year+phase)f(phase*season)	68	127703.124	249.428	0.000	8303.151
ϕ(phase+season)p(season*year+phase)f(phase*season)	63	127742.963	289.267	0.000	8353.054
ϕ(phase*season)p(season*year)f(phase*season)	66	127744.929	291.233	0.000	8348.986
ϕ(phase*season)p(season*year+phase)f(phase+season)	63	127759.503	305.807	0.000	8369.593
ϕ(phase+season)p(season*year)f(phase*season)	61	127792.149	338.454	0.000	8406.265
ϕ(phase*season)p(season*year)f(phase+season)	61	127810.159	356.464	0.000	8424.275
ϕ(phase+season)p(season*year+phase)f(phase+season)	58	127816.406	362.711	0.000	8436.550
ϕ(phase+season)p(season*year)f(phase+season)	56	127876.565	422.870	0.000	8500.735
ϕ(phase*season)p(season+year+phase)f(phase*season)	39	127882.804	429.109	0.000	8541.128
ϕ(phase*season)p(season+year)f(phase*season)	37	127910.730	457.034	0.000	8573.061
ϕ(phase+season)p(season+year+phase)f(phase*season)	34	127919.260	465.565	0.000	8587.611
ϕ(phase*season)p(season+year+phase)f(phase+season)	34	127934.580	480.885	0.000	8602.934
ϕ(phase+season)p(season+year)f(phase*season)	32	127959.767	506.072	0.000	8632.137
ϕ(phase*season)p(season+year)f(phase+season)	32	127977.127	523.432	0.000	8649.490
ϕ(phase+season)p(season+year+phase)f(phase+season)	29	128021.330	567.635	0.000	8699.711
ϕ(phase+season)p(season+year)f(phase+season)	27	128086.560	632.864	0.000	8768.956
